# A study of the MAYV replication cycle: Correlation between the kinetics of viral multiplication and viral morphogenesis

**DOI:** 10.1016/j.virusres.2022.199002

**Published:** 2022-11-10

**Authors:** Diogo C. Mendonça, Erik.V.S. Reis, Nídia.E.C. Arias, Hugo J. Valencia, Cláudio A. Bonjardim

**Affiliations:** Grupo de Transdução de Sinal, Laboratório de Vírus, Department of Microbiology, Institute of Biological Sciences, Universidade Federal de Minas Gerais., 31270-901, Avenida Antonio Carlos, 6627, Belo Horizonte, Minas Gerais, Brazil

**Keywords:** MAYV, Replication cycle, TEM, Timeline

## Abstract

Mayaro virus (MAYV) is mainly found in Central and South America and causes a febrile illness followed by debilitating arthritis and arthralgia similar to chikungunya virus (CHIKV). Infection leads to long-term sequelae with a direct impact on the patient's productive capacity, resulting in economic losses. Mayaro fever is a neglected disease due to the limited epidemiological data. In Brazil, it is considered a potential public health risk with the number of cases increasing every year. Most of our knowledge about MAYV biology is inferred from data obtained from other alphaviruses as well as more recent studies on MAYV. Here, we analyzed the kinetics of viral replication through standard growth curves, quantification of intracellular and extracellular particles, and RNA quantification. We compared transmission electron microscopy data during different stages of infection. This approach allowed us to establish a chronological order of events during MAYV replication and its respective timepoints including cell entry through clathrin-mediated endocytosis occurring at 15-30 min, genome replication at 2-3 h, morphogenesis at 4 hpi, and release at 4-6 hpi. We also present evidence of uncharacterized events such as ribosome reorganization as well as clusters of early viral precursors and release through exocytosis in giant forms. Our work sheds new and specific light on the MAYV replication cycle and may contribute to future studies on the field

## Highlights

• MAYV RNA synthesis starts at 2hpi, Morphogenesis at 3hpi and Liberation at 4hpi.
• Ribosome reorganization precedes early morphogenesis/liberation of particles.
• Clusters of nucleocapsid-like structures are related with early morphogenesis.
• CPV-II formation was correlated with exponential increase of virus particles.
• Timeline of events of MAYV replication was established.

## Graph Abstract

**Figure:**
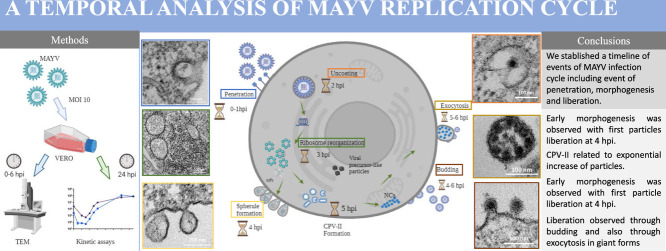

## 1 Introduction

The first report of Mayaro virus (MAYV) occurred in 1954 in Trinidad and Tobago where the virus was isolated from blood samples of rural workers in Mayaro County (Anderson et al., 1957). MAYV belongs to the realm *Riboviria*, kingdom *Orthornavirae*, phylum *Kitrinoviricota*, class *Alsuviricetes*, order *Martellivirales*, family *Togaviridae* and genus *Alphavirus* according to the international committee on taxonomy of viruses (ICTV) (Koonin et al., 2020). MAYV is classified under three genotypes: D (widely dispersed), L (limited), and N (new) with different geographic distributions (Auguste et al., 2015). The virus is transmitted mainly by the mosquito *Haemagogus janthinomys*, which circulates predominantly in Central and South America (Danillo Lucas Alves and Benedito Antonio Lopes da, 2018).

The alphavirus replication cycle starts with entry into the host cell through clathrin-mediated endocytosis (Carvalho et al., 2017; Helenius et al., 1980; Marsh et al., 1983). Inside the endosomes, the low pH leads to the uncoating and liberation of the viral genomic RNA in the cell (Helenius et al., 1980; Lescar et al., 2001), which is translated into nonstructural proteins that assemble a replication complex inside vesicular structures called spherules. In later stages of the cycle, these spherules are internalized inside large vacuoles called cytopathic vacuole I (CPV-I) (Spuul et al., 2010). Virions migrate to the cell membrane and are released through budding, thus gaining an envelope during the process (Ekström et al., 1994; Solignat et al., 2009; Suomalainen et al., 1992).

Despite knowledge about the alphavirus replication cycle, specific studies with MAYV are rare, and almost all of its cycle stages are proposed based on other alphavirus species such as Sindbis virus (SINV) and Chikungunya virus (CHIKV). The first ultrastructural studies of the MAYV replication cycle with transmission electron microscopy (TEM) were done in C6/36 and BHK-21 cells more than 30 years ago (Mezencio et al., 1990, 1989). More recently, Carvalho et al. (2017) showed that clathrin-mediated endocytosis is the main viral route of entry and is dependent on cholesterol-enriched caveolae-derived vesicles (Carvalho et al., 2017). Ribeiro-Filho et al. (2021) described the structure of mature MAYV particles obtained by cryo- electron microscopy (cryo-EM) (Ribeiro-Filho et al., 2021).

Here, our goal was to characterize the MAYV replication cycle in Vero cells through a temporal approach establishing timepoints of the main events involved in the cycle. We thus first determined the length of the cycle through plaque assays and then analyzed electron microscopy images of infected cells at different time points followed by comparison with data obtained from quantification of gRNA as well as intracellular and extracellular particles. We determined a timeline of observed events that correlate with our kinetic data including increased gRNA replication related to ribosome reorganization at 3 hpi, CPV-II formation related to exponential increase of immature particles at 5 hpi, and virus release through budding and exocytosis observed at 5 and 6 hpi.

## 2 Materials and methods

### 2.1 Cell culture and virus

Vero cells (ATCC CCL-81) were grown in Eagle's minimum essential medium (MEM) (Cultilab, São Paulo, Brazil) supplemented with 5% fetal bovine serum (FBS), antibiotics (200 U/mL penicillin and 40 µg/mL streptomycin), and antifungals (2 µg/mL amphotericin B) and incubated in a humidified atmosphere containing 5% CO_2_ at 37°C. The virus used in this work was MAYV genotype D isolate BeAr20290 (isolated in 1960 from a pool of *Haemagogus spp.*) The virus was kindly provided by Dr. Mauricio Lacerda Nogueira from the Faculty of Medicine of São José do Rio Preto (FAMERP, São José do Rio Preto, São Paulo, Brazil) and Dr. Cintia Lopes de Brito Magalhães from the Federal University of Ouro Preto (UFOP, Ouro Preto, Minas Gerais, Brazil).

### 2.2 Virus titration

The 1.5 × 10^5^ Vero cells/well were seeded in 24-well plates one day prior to MAYV infection. Viral samples were serially diluted 10-fold in MEM with 1% FBS and used to infect the cells. Next, 3 mL of semisolid 199 medium (Cultilab, São Paulo, Brazil) with 1% carboxymethylcellulose (CMC), 2% FBS, antibiotics (200 U/mL penicillin and 40 µg/mL streptomycin), and antifungals (2 µg/mL amphotericin B) was added to each well. Plates were incubated at 37°C for two days and then fixed overnight with 3.7% formaldehyde. Finally, the cells were washed with running water and stained with 1% crystal violet for 15 min. Visible plaque-forming units (PFU) were counted, and viral titers were determined (PFU/mL).

### 2.3 Virus growth curve kinetics

To generate growth curves, Vero cells were seeded in 24-well plates (approximately 1 × 10^5^ cells per well) 24 h prior to the infection. Cells were infected with MAYV at multiplicities of infection (MOI) of 10, 1, and 0.1 and incubated at 37°C with 5% CO_2_ for 1 hour for viral adsorption. The supernatant was then removed and replaced with 1 mL of MEM with 1% FBS. the supernatant was periodically collected and titrated.

To perform kinetic assays, Vero cells were infected under the same conditions as described above at an MOI of 10. The supernatants were analyzed through plaque assays to identify the beginning of viral release. At the beginning of virus morphogenesis and RNA replication, the supernatants were discarded, and the cell monolayer was washed with 1X PBS three times. Next, 300 µL of MEM was added to each cell monolayer and subsequently frozen and thawed three times, scraped, and collected in 1.5 mL microtubes and then centrifuged at 960 g for 5 min at 4°C. The supernatants were titrated for morphogenesis analysis or submitted to RNA extraction and RT-qPCR for RNA analysis.

### 2.4 RNA extraction and RT–qPCR

Total RNA was extracted from the cell monolayer using TRIzol reagent (Life Technologies). Here, 1 mL of TRIzol was added per well, and the samples were transferred to a 1.5 mL microcentrifuge tube after the rupture of the monolayer. The remainder of the protocol was performed according to the manufacturer´s instructions. Briefly, 120 µL of chloroform was added to each sample, and the sample was centrifuged after 10 min of incubation (12,000 g for 15 min at 4°C). The aqueous phase was then transferred to another 1.5 mL microcentrifuge tube, and 500 µL of isopropanol was added. After 10 min, the solution was centrifuged (12,000 g for 10 min at 4°C) and the flow-through discarded. The RNA was then resuspended in 1 mL of 75% ethanol, centrifuged (12000 g – 5 min – 4°C), and the flow-through discarded. Finally, the RNA was resuspended in 30 µL of Nuclease-free water.

The RNA samples were submitted to a RT–qPCR protocol using the Go-Taq 1-step RT-qPCR (Promega) reagent with primers targeting the nsP1 gene of MAYV genome: Forward 5’ CACGGACMTTTTGCCTTCA 3’; Reverse 5’ AGACTGCCACCTCTGCTKGAG 3’; and Probe 5’(VIC) ACAGATCAGACATGCAGG 3’ (Naveca et al., 2017). The reactions were performed using 500 nM of each primer and 200 nM of probe, 5 µL of Go-Taq master mix, reverse transcriptase (0.2 µL), and 3 µL of RNA samples all in a final volume of 10 µL. The amplification cycle was as follows: 1 cycle of 15 min at 45°C, 1 cycle of 2 min at 95°C, 40 cycles of 15 s at 95°C, and finally 60 s at 60°C. The Cycle threshold (CT) values were converted to fold changes and presented as arbitrary units: fold change x 10000.

### 2.5 Transmission electron microscopy

For TEM, Vero cells were infected as described in the previous section at an MOI of 10 and fixed each hour until 6 h post-infection with 2.5% glutaraldehyde in a 0.1 M sodium phosphate buffer for 2 h at room temperature. Cells were postfixed with 2% osmium tetroxide and embedded in Epon resin. Ultrathin sections were then analyzed under TEM (Spirit Biotwin FEI, 120 kV) at the Centre of Microscopy of Universidade Federal de Minas Gerais, Brazil.

### 2.6 Graphical design

Results from the kinetics experiment were analyzed with the GraphPad Prism 9.3.1 (GraphPad Software Inc., La Jolla, CA, USA). It was also used to plot the graphs and calculate averages and standard deviation.

## 3 Results

### 3.1 Mayaro virus growth curve kinetics and RNA synthesis

**Fig. 1:**
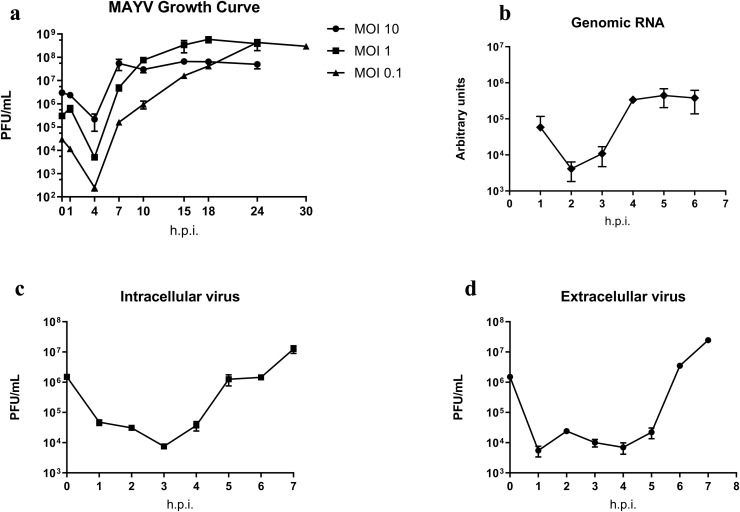
MAYV growth kinetics and RNA synthesis. (a) MAYV replication cycle: Vero cells were infected with MAYV at MOIs of 10, 1, and 0.1. The viral growth was monitored at different time points up to 24 hpi. The viral growth curves showed similar patterns with the eclipse phase at approximately 4 hpi followed by an exponential phase. (b–d) MAYV RNA replication and virus infectivity. Vero cells were infected with MAYV at an MOI of 10 and assessed at different time points. (b) The cell monolayer was collected and submitted to RNA extraction followed by qPCR. RNA replication started at approximately 2 hpi and plateaued at 4 hpi. (c, d) Viral infectivity from the cell monolayer or from the supernatant was determined by plaque assay. (c) Particle production started approximately 3–4 hpi. (d) Particle liberation started at approximately 5–6 hpi. Error bars indicate standard deviations from the means. Data is from two different experiments with two replicates each.

Our first step to better understand MAYV replication was to analyze the virus growth kinetics. We thus infected Vero cells at MOIs of 10, 1, or 0.1 and then determined the virus titer by plaque assay (Fig. 1a). The replication patterns for all analyzed conditions were similar: a fast cycle with an eclipse phase at approximately 4 hpi followed by an exponential increase in the number of particles that reached a plateau (stationary phase). The main differences were observed for the time required for the curve to reach the plateau, which occurred at 7 hpi for MOI 10 and 15 hpi for MOI 1; the corresponding maximum viral yield was 4.91 × 10^7^ and 3.85 × 10^8^, respectively. For a MOI 0.1 at 24 hpi, the curve did not seem to have plateaued, and thus we analyzed one additional timepoint at 30 hpi. However, the virus titer was of 2.98 × 10^8^, which is very close to the value from 24 hpi (4.3 × 10^8^) suggesting that the curve did plateau near 24 hpi.

We next investigated the kinetics of viral RNA synthesis, the production of infectious particles, and the liberation of virus particles in the supernatant. Vero cells were infected with MAYV at an MOI of 10, and the cell monolayer and the supernatant were then collected to determine viral infectivity (plaque assay) and RNA synthesis (RT-qPCR). Our data shows that the measurement of RNA decreased up to 2 hpi when synthesis started. This amount increased up to 82-fold at 4 hpi in the stationary phase (Fig. 1b). The determination of virus infectivity on the cell monolayer showed that the infectivity was also reduced up to 3 hpi when morphogenesis/maturation began, thus increasing thereafter up to 1,600 times at 7 hpi (Fig. 1c). The liberation of particles in the supernatant was verified at approximately 4 hpi and increased 1100 times at 7 hpi (Fig. 1d).

### 3.2 Mayaro virus penetration occurs until 1 HPI

To expand our knowledge concerning the MAYV replication cycle, we next used TEM to study the MAYV replication cycle: Vero cells were infected with MAYV at an MOI of 10, and images were collected 15 min post-infection (mpi), 30 mpi, and from 1 to 6 hpi in intervals of 1 hour and compared between each other and against the uninfected control (Supplemental Figure 1)

**Fig. 2:**
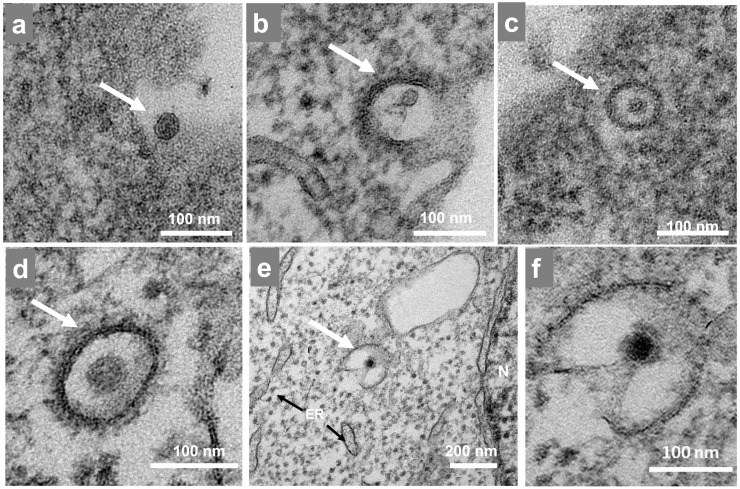
Mayaro virus cell entry. Vero cells were infected with Mayaro virus (MAYV) at an MOI of 10 and were assessed by TEM at (a) 15 mpi, (b, c) 30 mpi, and (d–f) 1.0 hpi. (a) A MAYV particle outside of the cell. (b) A MAYV particle undergoing clathrin-mediated endocytosis. (c, d) MAYV particle inside an endosome surrounded by clathrin proteins, and (e) Uncoating of a viral particle occurring on cell cytoplasm. (f) Magnification of image showed in panel E. White arrow indicate structure highlighted. Black arrows indicate cell organelles. N, nucleus; ER, endoplasmic reticulum. The images show electron micrographs with scale bars.

Our data show virus particles near the cell at 15 mpi (Fig. 2a) and entering the cell entry through membrane invagination to form an endocytic vesicle surrounded by clathrin proteins (Fig. 2b). By 30 mpi, the virus was observed inside an endosome (Fig. 2c-d) followed by uncoating of the particle through the fusion of the virus membrane with the endosome membrane (Fig. 2e-f).

### 3.3 Mayaro virus ribosome reorganization at 3HPI

**Fig. 3:**
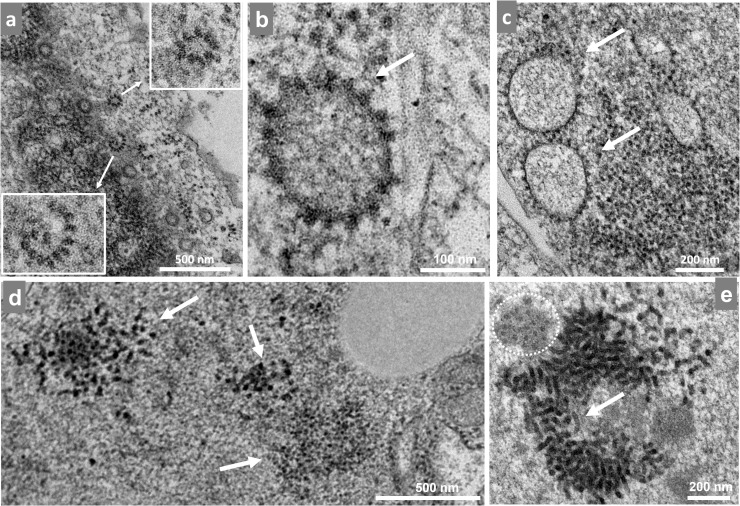
Mayaro virus induces ribosome reorganization. Vero cells infected with Mayaro virus (MAYV) at an MOI of 10 were assessed at 3 hpi by TEM. (a) Ribosomes were arranged in a circular pattern close to the nucleus with white rectangles showing a higher magnification. (b) Ribosomes were arranged in a circular pattern around a vacuole-like structure. (c) Giant cluster of ribosomes and three vacuole-like structures surrounded by nucleocapsids. (d) White arrows show cluster of nucleocapsid-like particles. (e) Two clusters of nucleocapsid-like particles—one is smaller (white dashed circle), and the other (white arrow)larger, which may represent a higher stage of development. The images show electron micrographs with scale bars.

The genomic RNA (gRNA) is directly translated to produce the nonstructural proteins (nsPs) nsP1-4 during the alphavirus replication cycle and after the uncoating of the virus leading to nucleocapsid disassembly. In turn, a replication complex is assembled that is responsible for gRNA production. The qPCR data indicated that genomic replication started between 2-3 hpi and was exponential by 4 hpi. When we observed our infected cell at this timepoint, there were many ribosomes (small dense spherical particles, 18–22 nm in size) distributed in the cytoplasm. These ribosomes were sometimes organized in a circular or semicircular pattern near large electrodense structures, which seemed to result from protein accumulation (Fig. 3a). We also observed ribosomes surrounding large vacuole-like structures; these were always as a single layer (Fig. 3b-c). Curiously, we also found dense and disorganized clusters of spherical particles that were larger than ribosomes and very similar to nucleocapsids that later appeared in the MAYV replication cycle (Fig. 3d). These particles were observed at different sizes and suggest different stages of development of these same particles; however, further studies are needed to understand and characterize this structure (Fig. 3e).

### 3.4 Mayaro virus-induced spherule formation at 4HPI

**Fig. 4:**
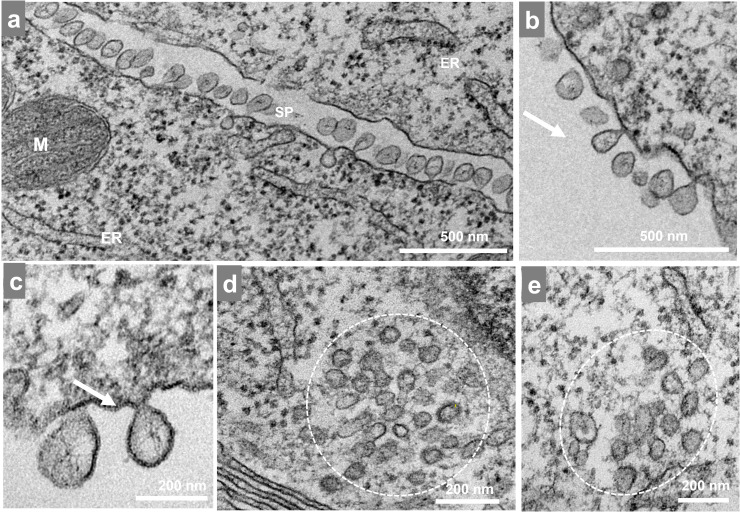
Mayaro virus-induced spherule formation. Vero cells infected with Mayaro virus (MAYV) at an MOI of 10 were assessed at 4 hpi by TEM. (a) Two infected cells with spherules between then. (b-c) Spherules outside and anchored to the cell. (d, e) Spherule-like structures inside the cell near the endoplasmic reticulum (white dotted area). ER, endoplasmic reticulum; M, mitochondria; SP, spherules The images show electron micrographs with scale bars.

RNA replication of alphaviruses occurs inside the vesicle compartments formed by nsPs directly translated from gRNA as well as denominated spherules (Hellström et al., 2017, Spuul et al., 2010). As previously mentioned, this ribosome reorganization directly correlated with the increase of RNA observed by qPCR at 3 hpi. The replication reached a plateau stage by 4 hpi—past this point we could no longer find these clusters of ribosomes. Rather, there were many spherules. These structures were observed both isolated and in groups, near—and in many cases anchored to—the cell membrane through a narrow neck (Fig. 4a-c). The size of the spherules was variable (50–100 nm) with circular and elliptic shapes. We also observed small groups of sphere-like structures inside the cytoplasm (Fig. 4d-e) usually near the endoplasmic reticulum (ER).

### 3.5 CPV-II formation at 5HPI and its correlation with Mayaro virus morphogenesis

**Fig. 5:**
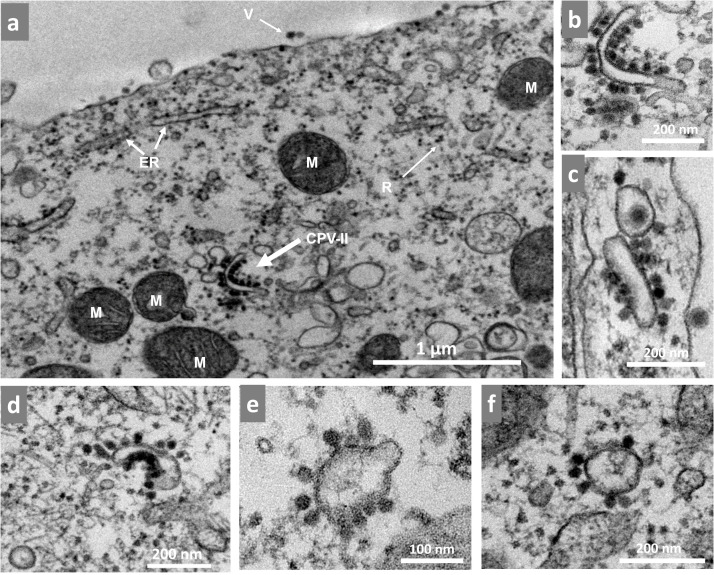
CPV-II formation correlates with Mayaro virus morphogenesis. Vero cells infected with Mayaro virus (MAYV) at an MOI of 10 were assessed 5–6 hpi by TEM. (a) Infected cell at 5hpi containing a CPV-II structure. (b), CPV-II observed in panel (a) magnified. (c-f) Other forms of CPV-II were also observed including (c) straight rods, (d) half-moon and (e, f) circular shapes. White arrow indicated structures. M, mitochondria; ER, endoplasmic reticulum; R, ribosomes; V, virus; CPV-II cytopathic vacuole type-II. The images show electron micrographs with scale bars.

The CPV-II of the alphavirus is a virus-induced structure derived from a trans-Golgi network containing E1/E2 glycoproteins with nucleocapsids (NCs) attached to its cytoplasmic side (Jose et al., 2017). Here, we confirmed the formation of CPV-II in Vero cells infected with MAYV at an MOI of 10 starting at 5 hpi (soon after the appearance of the first spherules). CPV-II was formed in the cytoplasm. It was isolated and of different shapes and sizes including curved rods (Fig. 5a-b), straight rods (Fig. 5c), half-moon (Fig. 5c) and circular shapes (Fig. 5d-e). These structures are usually surrounded on a single layer by a variable number of NCs similar to those previously described for CHIKV (Higashi et al., 1967; Reis et al., 2021). The timeline of these events was directly correlated with an exponential increase in the number of viruses collected from the cell monolayer, which is consistent with our data from kinetic assays (Fig. 1c).

### 3.6 Mayaro virus release

**Fig. 6:**
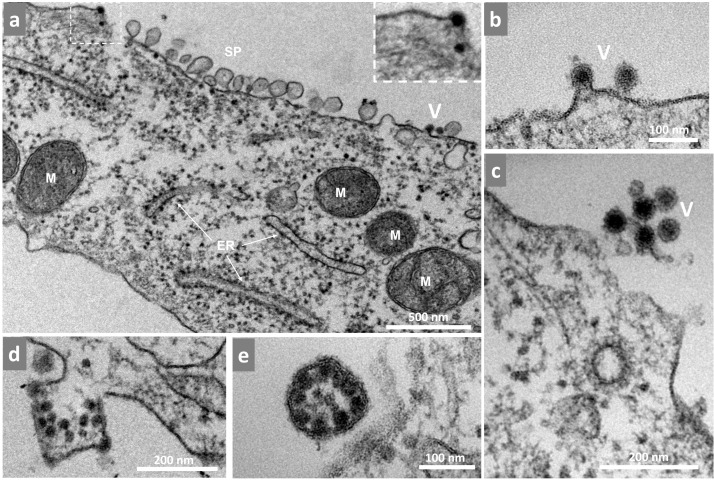
Mayaro virus release. Vero cells infected with Mayaro virus (MAYV) at an MOI of 10 were assessed 4–6 hpi by TEM. (a) - First budding particles observed at 4 hpi. (b) Particle on the left acquiring its envelope, and particle on the right already released. (c) Small cluster of particles released (d) Formation of a giant form containing viral particles at 5 hpi. (e) Giant form released at 6 hpi.; M, mitochondria; ER, endoplasmic reticulum; SP. Spherules; V, virus The images show electron micrographs with scale bars.

The classical Alphavirus way of particle release is via budding from the plasma membrane . We show that budding of MAYV particles began at 4 hpi although this was relatively rare and most of the released particles were observed after 5 hpi. On Fig. 6a we observe a cell with different viral particles including one on the left size undergoing budding. After 6 hpi the number of released particles observed increased even more (Fig. 6c). In addition to classical budding, we also observed the formation of large vesicles derived from the cell membrane at 5 hpi: These vesicles contained various particles. This structure was observed and still attached to the cell (Fig. 6d) with particles near its limiting membrane and budding. At 6 hpi, we observed another giant structure localized outside the cell and densely packed with viral particles (Fig. 6e). The timeline of these events was directly correlated with an exponential increase in the number of particles collected from the cell monolayer, which is consistent with our kinetic assays (Fig. 1c).

## 4 Discussion

There are few studies concerning MAYV biology—especially regarding the replication cycle on mammalian cells (Carvalho et al., 2017, Mezencio et al., 1990). We performed a temporal analysis of the first replication cycle of MAYV infection of Vero cells that occurred near 6–7 hpi. There were two procedures: (i) a kinetic analysis to determine genome replication by RT-qPCR and viral titration by plaque assay, and (ii) TEM to better characterize the steps of its replication cycle.

**Fig. 7:**
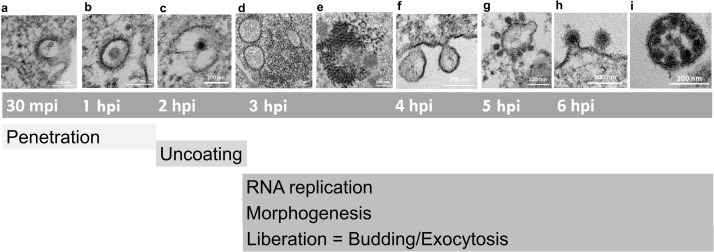
Timeline of events of the MAYV replication cycle. **(**a) Viral penetration starts at 15–30 mpi through clathrin-mediated endocytosis. (b) Particles are internalized into cell vesicles at 1 hpi. (c) The uncoating process begins at 2 hpi, and RNA replication begins. (d, e) At 3 hpi, morphogenesis of particles starts with d) reorganization of ribosomes and (e) formation of the first viral particles. (f) At 4 hpi, spherules were found in every cell, and the first particles began to bud from the cells. (g) At 5 hpi, CPV-II was abundant in the cytoplasm, and the number of particles increased exponentially. At 6 hpi, most of the particles left the cells either by (h) budding or by (i) exocytosis inside giant vesicles.

Based on these procedures, our study established a timeline of events of the MAYV replication cycle that correlated well with both our kinetic assays and ultrastructural analyses and what was partially described in the literature. See Fig. 7 for a summary of this temporal replication cycle description.

As verified with other alphaviruses (Göertz et al., 2017; Liu et al., 2018), MAYV presented a fast cycle with a short eclipse phase up to 4 hpi followed by exponential growth until it plateaued (Figure 1a).

MAYV entry occurs via the endocytic pathway, i.e., clathrin-mediated endocytosis as described for other alphaviruses (Marsh et al., 1983; Sourisseau et al., 2007) and via cholesterol-enriched caveolae-derived vesicles (Carvalho et al., 2017). Our micrographs showed that entry through endocytosis occurred between 15 and 30 mpi (Fig. 7a) including viral adsorption to the cell followed by penetration and transport inside vesicles at 1 hpi (Fig. 7b). The entry process is highly efficient (MOI 10), and the event occurred at high speed and was finalized within 30 mpi. This was followed by an eclipse phase (Fig. 1a) that included the uncoating process at 2 hpi (Fig. 7c). Carvalho et al., (2017) found similar timepoints of entry using fluorescence experiments with most particles inside the cells at 20–30 mpi (Carvalho et al., 2017).

At approximately 3 hpi (Fig. 7d), we found ribosomes organized in a circular shape around a vacuole-like structure (Fig. 3). We speculate that these structures localize where the translation of nonstructural proteins occurs preceding the spherule formation associated with the replication of viral RNA. In fact, spherules can be found in every cell at 4 hpi as previously described for other alphaviruses (Brown et al., 2018; Higashi et al., 1967) (Fig. 4). The spherules were anchored to the outer part of the cell membrane through a narrow neck where the replication complex is located and where the double-stranded RNA intermediary can evade degradation and recognition by pattern recognition receptors (Spuul et al., 2010). As the cycle progresses, spherules are internalized in large vacuoles forming CPV-1. At this stage of infection, we also observed many groups of internalized spherules (Fig. 4d-e) but did not find CPV-I structures.

Regarding morphogenesis, we first observed the formation of structures similar to viral precursors at 3 hpi (Fig. 7e) in densely packed groups (Fig. 3d-e). This is surprisingly early because we had still not observed the formation of spherules at the time. We did observe some actual particles at 4 hpi including some that were already budding, albeit scarce; we also observed spherules at this time (Fig. 7f). Finally, the kinetic data show the first increase in the number of particles on the cell monolayer from 3 to 4 hpi (Fig. 1c). These data suggest that these structures are indeed related to morphogenesis and may be related to an early and yet uncharacterized step in the viral replication cycle.

Soon after the formation of spherules (5 hpi; Fig. 7g), we started to observe the formation of CPV-II (Fig. 5). These structures are induced by alphavirus infection and contain viral glycoproteins in their vesicles. They are surrounded by nucleocapsids and can be found in both vertebrate and invertebrate cells (Jose et al., 2017). We observed different forms of CPV-II as previously described for other alphaviruses (Chen et al., 2013; Higashi et al., 1967; Reis et al., 2021). Previous studies with CHIKV described the appearance of CPV-II at the late stage of the replication cycle and occurring only after 24 hpi in invertebrate cells (Chen et al., 2013). However, in our previous study with CHIKV (Reis et al., 2021) as well as the present study both carried out with vertebrate cells we observed CPV-II at the early stages of the replication cycle. These differences may reflect the mechanisms of adaptation of the alphavirus to its different hosts.

The classical mechanism of alphavirus particle release is through budding, which we observed as early as 4 hpi and was more frequent after 6 hpi (Fig. 7h-i). We rarely observed these giant forms. These were structures derived from the plasma membrane containing immature particles. These giant forms were found either still attached to the cell or already released (Fig. 6d-e) and are an alternative method of viral release by exocytosis. These structures were described for some alphaviruses including CHIKV, which originates from mature CPV-II and is associated with the rapid release of a large number of viral particles in later stages of infection (Chen et al., 2013).

It is worth to note that with the experimental conditions used to characterize the MAYV replication cycle in this work (high MOI) we observed an overlap of events, mainly in the morphogenesis stages, and related to the formation of spherules and first particles. The use of a lower MOI could help to clarify these gaps; however, the low number of particles makes it difficult to observe events at early times, and at later times the multiple cycles has occurred already makings it impossible to determine the correct order of events.

## 5 Conclusions

This study organized the replication cycle of MAYV from entry to release in a chronological order and assigned timepoints to different phases including penetration, RNA replication, morphogenesis, and liberation. We also identified events related to uncoating, ribosome reorganization, particle formation, budding, and exocytosis. We note some undescribed events of MAYV replication including early morphogenesis preceding spherule formation, absence of CPV-I, and release through giant forms. We expect that this study will expand our current knowledge concerning MAYV replication and offer insight on viral biology and contribute to further studies on the field

## Data Availability

Viral samples are available upon request.

## Supplementry

**Supplemental Figure 1:** Uninfected cells Micrographs of healthy uninfected cells (a-c). N, nucleus; ER, endoplasmic reticulum. M. mitochondria; G, Golgi apparatus. The images show electron micrographs with scale bars.

## CRediT authorship contribution statement

**Diogo C. Mendonça:** Conceptualization, Investigation, Methodology, Writing – original draft, Writing – review & editing. **Erik.V.S. Reis:** Methodology, Investigation. **Nídia.E.C. Arias:** Methodology, Investigation. **Hugo J. Valencia:** Conceptualization, Methodology. **Cláudio A. Bonjardim:** Conceptualization, Funding acquisition, Supervision, Writing – review & editing.

## Declaration of Competing Interest

The authors declare that they have no known competing financial interests or personal relationships that could have appeared to influence the work reported in this paper.

## Data Availability

Data will be made available on request.

## Appendix Supplementary materials

Image, image 1

Image, image 2

## References

[bib0001] Anderson C.R., Downs W.G., Wattley G.H., AHIN N.W., Reese A.A. (1957). Mayaro virus: a new human disease agent. II. Isolation from blood of patients in Trinidad, B.W.I. Am. J. Trop. Med. Hyg..

[bib0002] Auguste A.J., Liria J., Forrester N.L., Giambalvo D., Moncada M., Long K.C., Morón D., de Manzione N., Tesh R.B., Halsey E.S., Kochel T.J., Hernandez R., Navarro J.-C., Weaver S.C. (2015). Evolutionary and ecological characterization of Mayaro virus strains isolated during an outbreak, Venezuela, 2010. Emerg. Infect. Dis..

[bib0003] Brown R.S., Wan J.J., Kielian M. (2018). The alphavirus exit pathway: what we know and what we wish we knew. Viruses.

[bib0004] Carvalho C.A.M., Silva J.L., Oliveira A.C., Gomes A.M.O. (2017). On the entry of an emerging arbovirus into host cells: Mayaro virus takes the highway to the cytoplasm through fusion with early endosomes and caveolae-derived vesicles. PeerJ.

[bib0005] Chen K.C., Kam Y.-W., Lin R.T.P., Ng M.M.-L., Ng L.F., Chu J.J.H. (2013). Comparative analysis of the genome sequences and replication profiles of chikungunya virus isolates within the East, Central and South African (ECSA) lineage. Virol. J..

[bib0006] Danillo Lucas Alves E., Benedito Antonio Lopes da F. (2018). Characterization of the immune response following in vitro mayaro and chikungunya viruses (Alphavirus, Togaviridae) infection of mononuclear cells. Virus Res.

[bib0007] Ekström M., Liljeström P., Garoff H. (1994). Membrane protein lateral interactions control Semliki Forest virus budding. EMBO J..

[bib0008] Göertz G.P., Vogels C.B.F.F., Geertsema C., Koenraadt C.J.M.M., Pijlman G.P. (2017). Mosquito co-infection with Zika and chikungunya virus allows simultaneous transmission without affecting vector competence of Aedes aegypti. PLoS Negl. Trop. Dis..

[bib0009] Hellström K., Kallio K., Utt A., Quirin T., Jokitalo E., Merits A., Ahola T. (2017). Partially uncleaved alphavirus replicase forms spherule structures in the presence and absence of RNA template. J. Virol..

[bib0010] Higashi N., Matsumoto A., Tabata K., Nagatomo Y. (1967). Electron microscope study of development of Chikungunya virus in green monkey kidney stable (VERO) cells. Virology.

[bib0012] Jose J., Taylor A.B., Kuhn R.J. (2017). Spatial and temporal analysis of alphavirus replication and assembly in mammalian and mosquito cells. mBio.

[bib0013] Koonin E.V., Dolja V.V., Krupovic M., Varsani A., Wolf Y.I., Yutin N., Zerbini F.M., Kuhn J.H. (2020). Global organization and proposed megataxonomy of the virus world. Microbiol. Mol. Biol. Rev. MMBR.

[bib0014] Lescar J., Roussel A., Wien M.W., Navaza J., Fuller S.D., Wengler Gisela, Wengler Gerd, Rey F.A. (2001). The fusion glycoprotein shell of Semliki forest virus: an icosahedral assembly primed for fusogenic activation at endosomal pH. Cell.

[bib0015] Liu X., Mutso M., Utt A., Lepland A., Herrero L.J., Taylor A., Bettadapura J., Rudd P.A., Merits A., Mahalingam S. (2018). Decreased virulence of ross river virus harboring a mutation in the first cleavage site of nonstructural polyprotein is caused by a novel mechanism leading to increased production of interferon-inducing RNAs. mBio.

[bib0016] Marsh M., Bolzau E., Helenius A. (1983). Penetration of semliki forest virus from acidic prelysosomal vacuoles. Cell.

[bib0017] Mezencio J.M., de Souza W., Fonseca M.E., Rebello M.A. (1990). Ultrastructural study of Mayaro virus replication in BHK-21 cells. Arch. Virol..

[bib0018] Mezencio J.M., de Souza W., Fonseca M.E., Rebello M.A. (1989). Replication of Mayaro virus in Aedes albopictus cells: an electron microscopic study. Arch. Virol..

[bib0019] Naveca F.G., do Nascimento V.A., de Souza V.C., Nunes B.T.D., Rodrigues D.S.G., da Costa Vasconcelos P.F. (2017). Multiplexed reverse transcription real-time polymerase chain reaction for simultaneous detection of Mayaro, Oropouche, and oropouche-like viruses. Mem. Inst. Oswaldo Cruz.

[bib0020] Reis E.V.S., Damas B.M., Mendonça D.C., Abrahão J.S., Bonjardim C.A. (2021). In-depth characterization of the chikungunya virus replication cycle. J. Virol..

[bib0021] Ribeiro-Filho H.V., Coimbra L.D., Cassago A., Rocha R.P.F., Guerra J.V., da S., de Felicio R., Carnieli C.M., Leme L., Padilha A.C., Paes Leme A.F., Trivella D.B.B., Portugal R.V., Lopes-de-Oliveira P.S., Marques R.E. (2021). Cryo-EM structure of the mature and infective Mayaro virus at 4.4 Å resolution reveals features of arthritogenic alphaviruses. Nat. Commun..

[bib0023] Solignat M., Gay B., Higgs S., Briant L., Devaux C. (2009). Replication cycle of chikungunya: a re-emerging arbovirus. Virology.

[bib0024] Sourisseau M., Schilte C., Casartelli N., Trouillet C., Guivel-Benhassine F., Rudnicka D., Sol-Foulon N., Roux K.L., Prevost M.-C., Fsihi H., Frenkiel M.-P., Blanchet F., Afonso P.V., Ceccaldi P.-E., Ozden S., Gessain A., Schuffenecker I., Verhasselt B., Zamborlini A., Saïb A., Rey F.A., Arenzana-Seisdedos F., Desprès P., Michault A., Albert M.L., Schwartz O. (2007). Characterization of reemerging chikungunya virus. PLoS Pathog..

[bib0025] Spuul P., Balistreri G., Kääriäinen L., Ahola T. (2010). Phosphatidylinositol 3-kinase-, actin-, and microtubule-dependent transport of semliki forest virus replication complexes from the plasma membrane to modified lysosomes. J. Virol..

[bib0026] Suomalainen M., Liljeström P., Garoff H. (1992). Spike protein-nucleocapsid interactions drive the budding of alphaviruses. J. Virol..

