# Supplementary figures and images for: A study of the MAYV replication cycle: Correlation between the kinetics of viral multiplication and viral morphogenesis
Source: Virus Res. 2022 Nov 10;323:199002. doi: 10.1016/j.virusres.2022.199002 (PMC10194297; doi:10.1016/j.virusres.2022.199002)

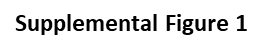

Supplement: Supplementary file 1 [file mmc1.zip › mmc1.png]

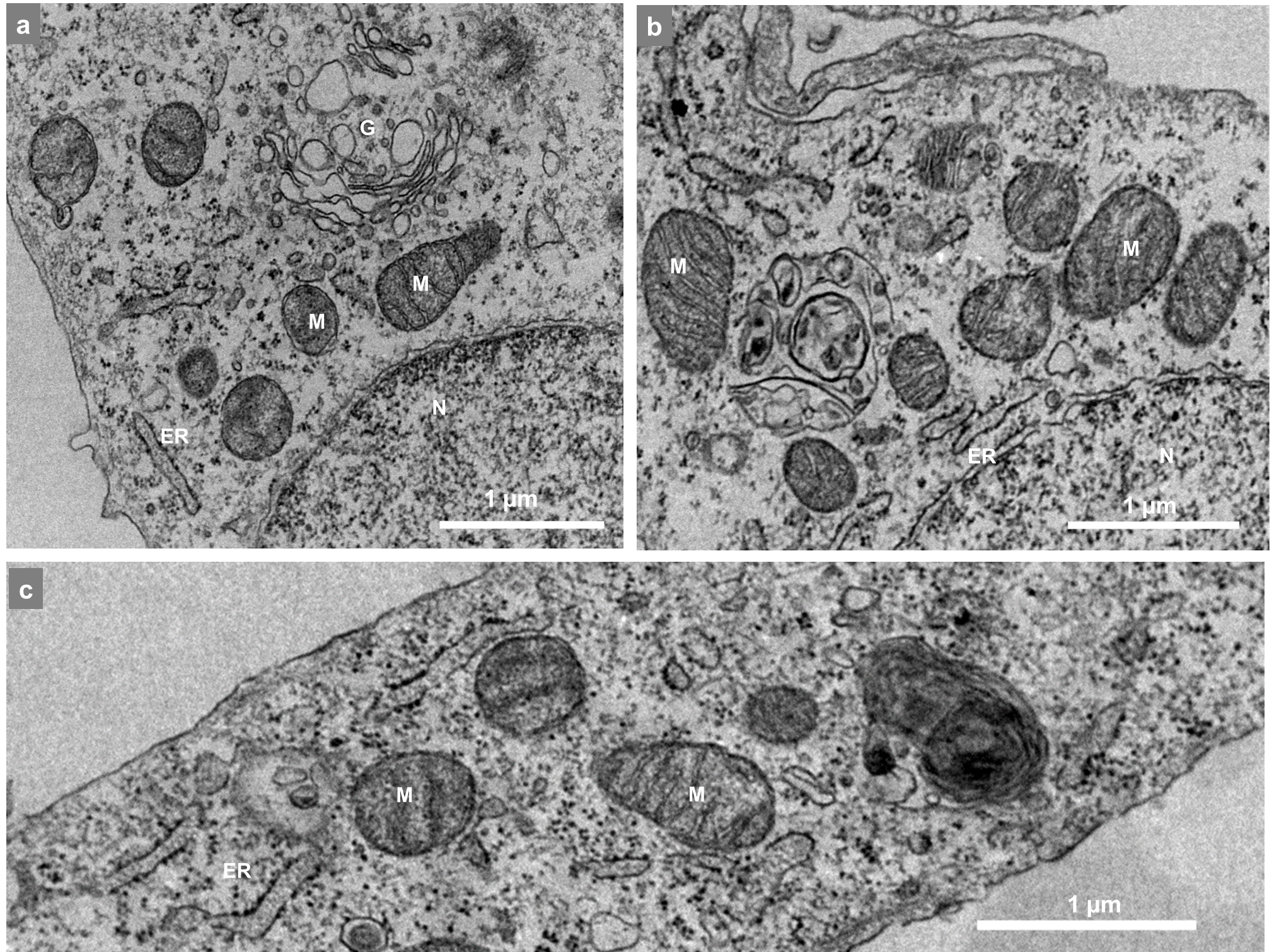

Supplement: Supplementary file 2 [file mmc2.zip › mmc2.tif]
